# Hurricanes and Health Equity: A Review of Structural Determinants of Vulnerability for Climate and Health Research

**DOI:** 10.1007/s40572-025-00475-w

**Published:** 2025-02-01

**Authors:** Shifali Mathews, Genee Smith, Jaime Madrigano

**Affiliations:** Department of Environmental Health and Engineering, Johns Hopkins Bloomberg School of Public Health, Baltimore, MD USA

**Keywords:** Hurricane vulnerability, Health equity, Climate change, Structural determinants of health, Structural racism

## Abstract

**Purpose of Review:**

Understanding hurricane vulnerability is crucial for targeting and identifying climate adaptation measures. However, vulnerability assessments often focus on proximal factors, which may obscure underlying drivers of health inequities. We sought to describe the literature characterizing hurricane vulnerability in the U.S., from 2000 to 2022. We abstracted the approaches and factors in each hurricane vulnerability assessment study, and developed a conceptual framework to guide data collection on structural determinants of climate vulnerability.

**Recent Findings:**

The review included a total of 121 studies. The majority pre-specified vulnerable populations, while 40% empirically derived vulnerability. Downstream factors pertaining to demographics, spatial analysis, and health status were most commonly used to assess vulnerability to hurricanes. Only five studies reported structural vulnerabilities, including racism, governance, institutions, and infrastructure deficiencies, which form the basis of our conceptual framework.

**Summary:**

Most hurricane vulnerability studies do not consider upstream factors of health inequities. We developed a conceptual framework and provided example data measures for structural determinants to incorporate into climate and health research, facilitating the development of more effective interventions to address root causes.

**Supplementary Information:**

The online version contains supplementary material available at 10.1007/s40572-025-00475-w.

## Introduction

The public health impacts of hurricanes and floods are substantial and several trends such as an increase in the frequency and severity of severe weather events and population growth in high-risk areas (e.g., coastal waterfronts) are expected to exacerbate this growing public health crisis [1–3]. Adverse impacts to population health can occur through exposure to floodwaters and mold, delayed access to medications and worsening chronic conditions, disorganized or unheeded evacuations, post-event infrastructure damage such as utility outages, and the experience of personal trauma through the loss of loved ones, homes, or economic livelihoods [4, 5]. Evidence indicates that such exposures lead to increases in morbidity and mortality due to injuries; cardiovascular, respiratory, renal, and infectious disease causes; and mental health disturbances [4, 6].

It has long been recognized that population vulnerability to the health impacts of hurricanes is a product of the inter-relationship between the magnitude of the exposure hazard, existing personal and place-based vulnerabilities, and social conditions [7–9]. Numerous studies have demonstrated that individuals living in low-income households and members of racially and ethnically minoritized groups suffer more severe consequences from hurricanes [10–12]. However, it is unclear if such population vulnerability stems from a greater sensitivity to hurricane exposure (e.g., because of greater prevalence of underlying chronic conditions or other chronic stressors) or from greater exposure to the hazard (e.g., low-income and racially and ethnically minoritized groups are more likely than others to live in neighborhoods that are prone to flooding) [13]. Further, a focus on disparities in exposure and outcomes can serve to call attention to a problem but may also obscure the underlying drivers of health inequities and, thus, take the focus away from the systemic interventions needed to improve health equity.

To call attention to these underlying drivers of health inequities and improve public health outcomes, there is an emerging discourse prioritizing the need to address *structural* determinants of health at the federal, public, and private levels [14]. According to the American Academy of Family Physicians (AAFP), the structural determinants of health are “the social, economic, and political mechanisms which generate social class inequalities in society” [15]. Key examples include governance, cultural and societal values, institutional practices, structural racism, and macroeconomic, social, and public policies [16, 17]. In the United States, the theoretical underpinnings of structural determinants of health have been extensively explored in the context of specific health domains (e.g., maternal and child health) [18, 19]. Here, we seek to expand this discussion to the climate change and health context.

We focus on hurricanes, one type of climate-related event, as a case study because there are direct and indirect pathways from their impacts that lead to health disparities, such as increased risk of injury or death, displacement and housing instability, and disruptions in healthcare access [20], all of which may be influenced by structural determinants. Appropriately identifying and measuring an upstream driver (structural determinant) of health can reveal opportunities for intervention that address the root causes of both existing and emerging health disparities, underscoring the interconnectedness of other upstream structural factors that shape health outcomes [21]. As such, we use the terms “structural determinants of health” and “structural determinants of vulnerability” interchangeably in this review. Within the context of hurricanes and climate change, socially vulnerable groups (e.g., racially and ethnically marginalized and low-income communities) face a syndemic of structural factors that exacerbate social and health inequalities, demonstrated by disproportionate residence in flood zones, pre-existing chronic conditions, limited ability to evacuate, and inadequate health and flood insurance [22].

The objectives of this review are to (1) characterize the literature on hurricane-related health vulnerability with a particular interest in understanding the extent to which structural determinants of health have been assessed; (2) situate the results of the review within the broader literature on structural determinants of health to develop a conceptual framework that considers structural determinants of climate and health vulnerability; and (3) develop recommendations to advance the field through consideration of structural determinants in the analysis of health vulnerability to hurricanes and other climate-related disasters.

## Methods

We conducted a literature search in the National Library of Medicine’s MEDLINE/PubMed database (https://pubmed.ncbi.nlm.nih.gov/). The inclusion criteria were designed to identify population-based research studies that assessed equity or vulnerability within the context of hurricane-related health impacts in the U.S. and Puerto Rico. In order to capture some of the most devastating hurricanes in recent history, the search covered articles published from January 2000 to October 2022. Our search criteria included terms to capture the type of event (hurricane OR “tropical cyclone”) and terms to focus on equity (equit* OR vulnerab*). We included “tropical cyclone” in the search strategy to be comprehensive, as it represents a broader weather phenomenon of which hurricanes are an example. Hurricanes and typhoons are types of strong tropical cyclones but are region-specific [23]. These terms are often used interchangeably and refer to an independent, large-scale, low-pressure system over tropical or subtropical waters characterized by organized thunderstorm activity and a clearly defined cyclonic wind pattern at the surface [23]. Although our search terms were intended to be comprehensive, we use the standard term “hurricane” for the remainder of this review, which encompasses our region of interest (i.e., the North Atlantic Ocean, the Northeast Pacific Ocean east of the dateline, or the South Pacific Ocean) in the United States and Puerto Rico [23, 24]. The search was not limited to any specific health outcome.

Reasons for exclusion were that the article did not include health impacts, focused broadly on population health impacts but did not examine hurricane-related vulnerability or equity, described conceptual models, were based outside of the U.S. and Puerto Rico, or were formats which were outside of the scope of this analysis (e.g., book, editorial). Studies that included outcomes relevant to hurricanes but not necessarily health outcomes were excluded from analysis. However, studies that indirectly lead to morbidity, mortality, or a disruption in healthcare services (e.g., infrastructure damage, flood risk) were included.

For each included study, information was abstracted on the type of vulnerability assessment category, the vulnerability factors considered, health outcomes, and the event. Two individuals (S.M. and J.M.) abstracted this information and reviewed the other’s analysis. The reviewers discussed discrepancies in the data abstraction until consensus was reached.

We additionally explored conceptual frameworks on health equity and structural determinants of health in both climate and broader public health contexts. This information informed the development of our conceptual framework, which connects the structural determinants of health related to hurricane vulnerability that we found in the literature to pathways leading to health disparities.

## Results & Discussion

**Fig. 1:**
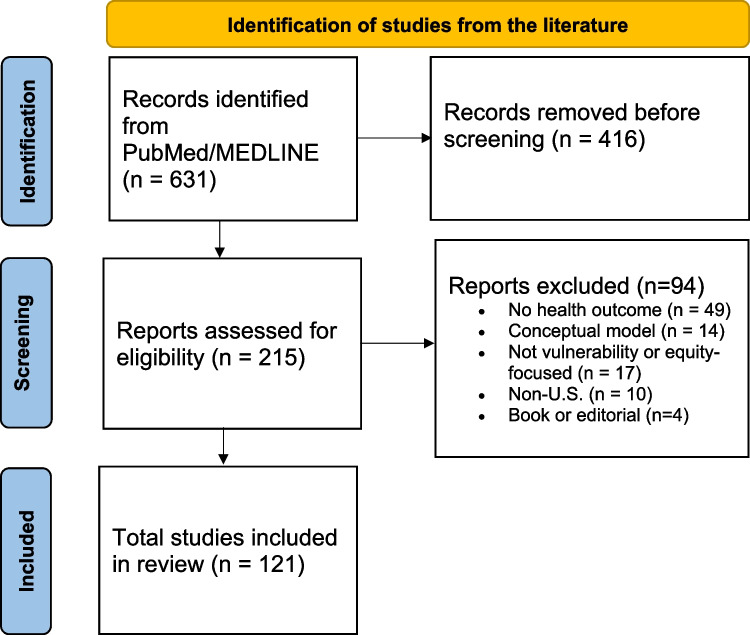
PRISMA diagram for identification of studies from the literature

The initial screen resulted in a large number of articles (*n* = 631), for which titles were reviewed for relevance. After completion of the title screening, the remaining articles (*n* = 215) were further screened through a review of abstracts and full-text, and an additional set (*n* = 94) were excluded (Fig. 1). Ineligible studies and their reasons for exclusion are also reported in Fig. 1. The remainder of this article focuses on lessons learned from the final set of papers reviewed (*n* = 121) (Supplementary Information).

The reviewed studies cover a wide geographic range, which we inferred by capturing the hurricane event under study (Supplemental Information). Hurricane Sandy (*n* = 32) occurred in the Northeastern U.S. in 2012. Within the U.S. Gulf Coast, Hurricane Katrina (*n* = 23) had catastrophic effects in 2005, followed by Hurricane Harvey in 2017 (*n* = 15), Hurricane Rita in 2005 (*n* = 2), and Hurricane Ike in 2008 (*n* = 1). In Puerto Rico, Hurricane Maria (*n* = 14) and Hurricane Irma (*n* = 11) caused widespread damage in 2017, with Irma also impacting Florida. Finally, Hurricane Florence (*n* = 3) affected the Carolinas in 2018. Some studies examined multiple of these hurricane events, while 21 separate studies did not identify a specific hurricane event.

**Fig. 2:**
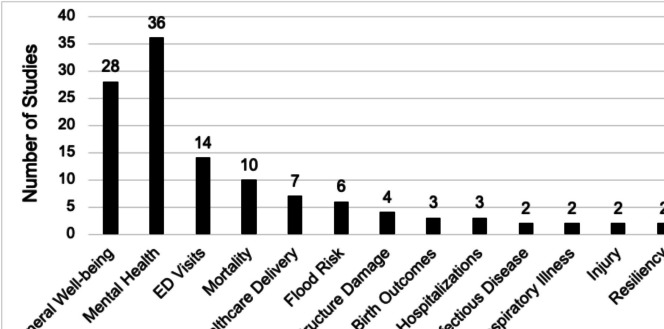
Outcomes examined in studies assessing equity or vulnerability within the context of hurricanes in the U.S. and Puerto Rico. The figure does not present sum totals to provide a comprehensive visualization, as a single study could focus on multiple health outcomes

Across the final set of reviewed papers, a variety of health outcomes were assessed (Fig. 2). The most commonly examined health outcomes were mental health (*n* = 36), general health and well-being (*n* = 28), and emergency department visits (*n* = 14). Other frequently assessed health outcomes included mortality (*n* = 10) and healthcare delivery (*n* = 7).

### Current Landscape of Hurricane Vulnerability Assessments

Broadly defined, there are two ways that vulnerability to hurricanes is considered in the reviewed literature: (1) through an empirically-based data analysis; or (2) through a theoretical framework, which specifies a group or context within which higher vulnerability is expected, and as such, a focused assessment is performed for that group or context. The majority of the reviewed studies (*n* = 71) used the latter approach with pre-specified populations or contexts of interest. The remaining reviewed studies included an empirical derivation of vulnerability (*n* = 48) or both types of assessments (*n* = 2).

**Fig. 3:**
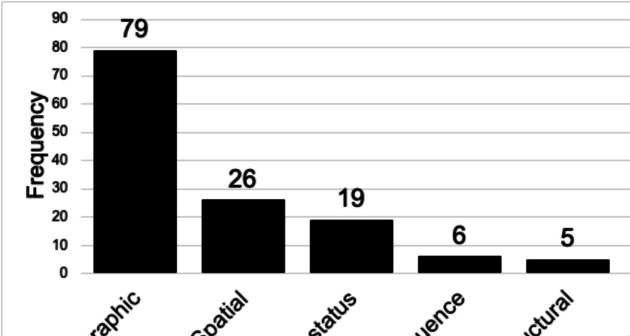
Breakdown of reviewed articles by type of vulnerability assessment category. The figure does not present sum totals to provide a comprehensive visualization, as a single study could report multiple factors of vulnerability

Within both types of studies (empirically-based and pre-specified), there are common categories by which researchers consider vulnerability, with some overlap: demographic (*n* = 79), spatial/geographic (*n* = 26), and health status (*n* = 19). Less common categories were consequence (*n* = 6), structural (*n* = 5), and occupational (*n* = 2) (Fig. 3). Age, race/ethnicity, sex/gender, and socioeconomic status (SES) were the most common factors for demographic-based vulnerability assessments. Populations characterized by health status included those on dialysis, end-stage renal disease (ESRD), diabetes, medically fragile, impairments/disabilities, and people living with HIV. The most common metrics for spatial data were based on county and census tract-level data. Furthermore, two main consequences of hurricane vulnerability were identified: displacement and exposure to toxic floodwaters. Occupationally vulnerable populations included first responders and health and social care workers.

Some of the reviewed studies had a combination of vulnerability assessment types. Out of the studies that assessed vulnerability demographically and spatially (*n* = 9), the factors included SES (*n* = 6), race/ethnicity (*n* = 3), county (*n* = 3), age (*n* = 2), high impact area (*n* = 2), census tract (*n* = 2), sex/gender (*n* = 1), and borough (*n* = 1). Demographic and health status assessments (*n* = 4) explored vulnerability by SES (*n* = 2), race/ethnicity (*n* = 1), age (*n* = 1), substance use (*n* = 1) general health susceptibility (*n* = 1), medical fragility (*n* = 1), and existing mental health condition (*n* = 1). Fitzpatrick et al. conducted a vulnerability assessment based on demographics and consequences and investigated race/ethnicity, age, and displacement [25]. Kelman et al. (2015) focused on ESRD patients in a high impact area (i.e., Sandy-affected area) through a health and spatial vulnerability assessment [26].

### Structural Determinants of Hurricane Vulnerability from Literature Review

In the included studies, only 4% (*n* = 5) investigated structural determinants of vulnerability, according to the AAFP definition and prior literature [15, 16, 27, 28]. Structural determinants considered in these papers were racism, governance, institutions, and infrastructure. While our search only yielded a limited number of studies on structural determinants of hurricane vulnerability, it is important to highlight how these upstream factors engender current health disparities within the context of hurricanes and other climate-related disasters, in order to inform future research and interventions. Below, we provide more detail on each of these structural determinants, how they were measured within the context of the reviewed studies, and their contribution to hurricane vulnerability.

#### Governance

Tennant and Gilmore (2020) explored the spatial relationship between government effectiveness and tropical cyclone mortality in 67 countries (including the U.S.) using negative binomial regression models [29]. Annual country-level scores for government effectiveness, established by the World Governance Indicators (WGI), were used to represent government effectiveness through public policy and service delivery. Real GDP per capita, primary school enrollment, and other institutional factors (i.e., infant mortality and settlements of politically excluded ethnic groups) were other development indicators of vulnerability that served as good predictors of tropical cyclone mortality. These factors reflect weak institutional capacity and national programs that do not capture vulnerable populations, resulting in more severe impacts from tropical cyclones in these areas [29]. Although comparisons are made at the country-level, it provides a useful framework for thinking about government effectiveness and other institutional factors that can influence tropical cyclone mortality and can potentially be measured at more granular levels.

#### Infrastructure

Aging and inadequate infrastructure influences pre-existing vulnerability, and damage to this infrastructure is a major barrier to disaster response, recovery, and resilience. Guerra Velázquez (2022) investigated this relationship in Puerto Rico after Hurricane Maria by conducting a gray literature search and interviews with public health professionals and residents [30]. Prior to Hurricane Maria, infrastructural deficiencies in electric grids and water and sewage systems stemming from an economic recession and related budget cuts led to health disparities in cancer and leptospirosis in the Puerto Rican population, which were exacerbated following the hurricane. Hurricane Maria created additional disparities in the areas of healthcare delivery, mental health, access to clean water, education, drownings, and mortality due to widespread power outages, especially for those who could not afford generators and for those living in flood zones [30]. Other challenges that contributed to disparate health outcomes were delays in reconstruction, recovery, and communication (i.e., cell phone towers) [30]. Children were particularly vulnerable to food and water shortages, damage to homes, and educational displacement [30].

Similarly, Ruiz Aviles et al. (2022) explored the resilience and adaptation of Puerto Rico’s community drinking water systems, which continued to provide services despite widespread failures after Hurricanes Irma and Maria, through a mixed-methods study design [31]. Interviews with drinking water system regulators (i.e., water system managers, government, and nongovernmental organization (NGO) representatives) were conducted, in addition to surveying water system managers and a gray literature review of newspaper articles [31]. Drinking water systems were poorly structured before both hurricanes due to financial and elevation constraints, and nearly half of these failed to meet water quality requirements [31]. Results from this exploratory case study reveal only a small reduction in non-compliance, increases in service interruption, and a significant energy cost burden following Hurricanes Irma and Maria [31]. While community adaptation allowed for water system functioning and eventual stabilization, there are organizational and financial barriers that limit the robustness of these drinking water systems (e.g., water quality) before, during, and after disasters [31].

#### Institutions

Public institutions are another example of structural determinants of vulnerability. One particular institution is the U.S. prison system, which houses a vulnerable population who lack overall freedom and have been overlooked in the disaster management process [32]. A cross-case synthesis of secondary qualitative data from two correctional facilities during Hurricanes Katrina and Maria found that evacuation and relocation standards were not followed adequately during these hurricane situations, along with limited local government oversight [32]. There are key areas for improvement in hurricane and emergency preparedness for correctional facilities involving evacuation practices and humane treatment.

#### Racism

Racism was a structural determinant that was analyzed in the context of emergency response after natural disasters, particularly through the effects of colonialism in Puerto Rico after Hurricanes Maria and Irma [33]. Both race and racism were measured through semi-structured qualitative interviews with community members who actively participated in resulting emergency response and relief efforts [33]. Interviewees revealed that systemic racism was a barrier for emergency and recovery response to natural disasters through issues with political status, federal and international aid, and inhibitions to community philanthropy [33].

### Conceptual Framework

**Fig. 4:**
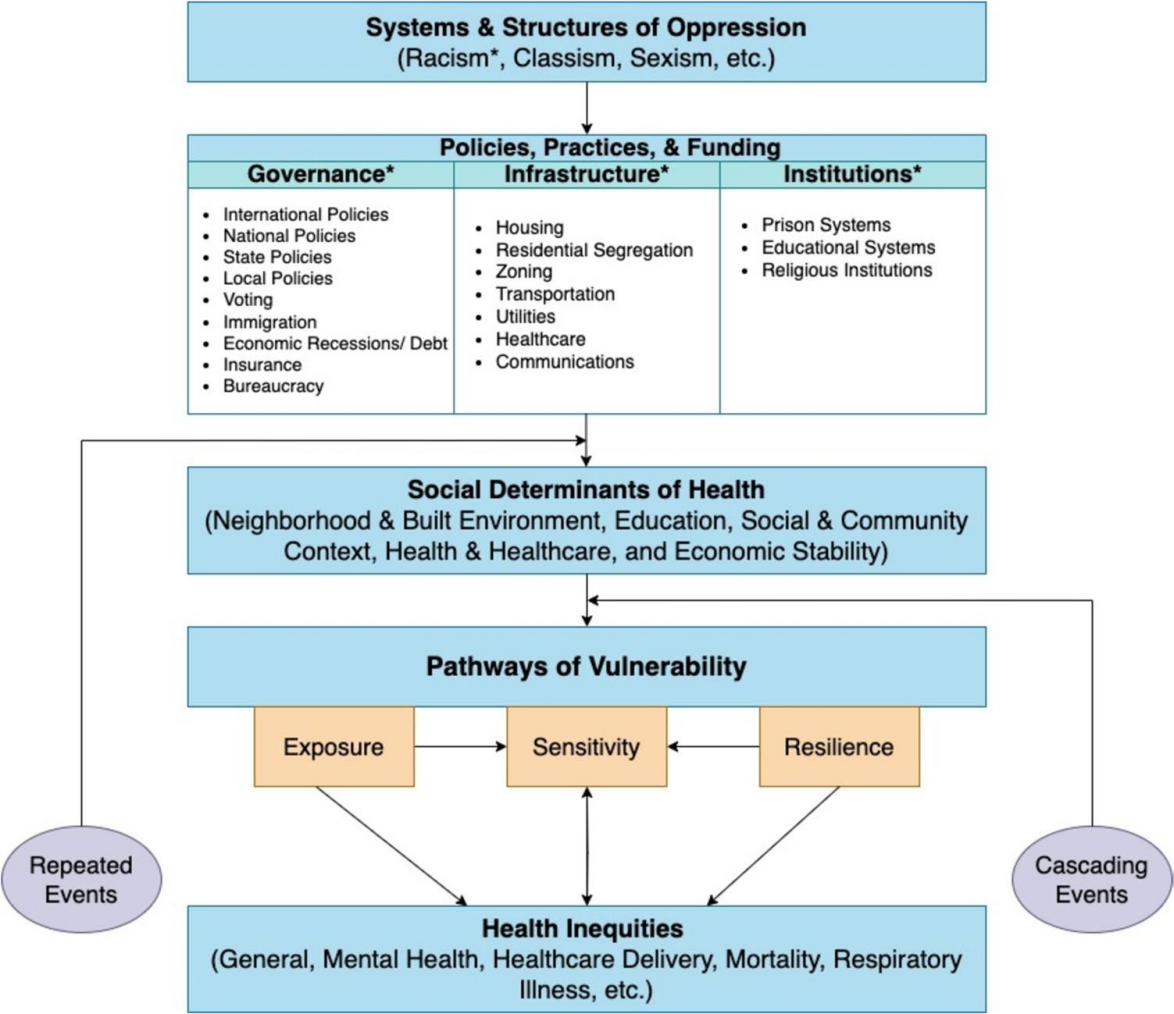
Conceptual framework on the impacts of structural determinants of health on hurricane vulnerability and resultant health inequities, building on existing frameworks by Smith et al., 2020 [27] and Yearby, 2020 [28]. *indicates one of the structural determinants of hurricane vulnerability identified in the search strategy/ literature review

To develop our conceptual framework (Fig. 4), we first reviewed several frameworks focused on climate justice and health equity. Smith et al. (2022) and others propose that socially vulnerable populations (i.e., low-income individuals and racially minoritized communities) experience structural inequalities and environmental injustices through policies, practices, and funding, which then lead to health inequities through disparities in exposure, sensitivity, and resilience at different stages of a climate disaster (i.e., before, during, and after the event) [27, 34]. Yearby (2020) details further how structural discrimination impacts health disparities, mediated by laws and the social determinants of health [28]. A combination of structural factors generates differences in social determinants of health at various geographic scales [35]. On an international scale, the World Health Organization’s (WHO) social determinants of health framework identify structural factors as drivers of socioeconomic status and capital, as well as contributors to differences in mediating factors (e.g., living conditions, psychosocial influences, and behaviors) [16]. On a national scale, the National Institute for Minority Health and Health Disparities’ (NIMHD) research framework identifies domains (biological, behavioral, physical/built environment, sociocultural environment, and healthcare system) and levels (individual, interpersonal, community, and societal) of influence for health disparities [36]. Furthermore, on a local scale, urban sustainability and resilience planning requires a socioecological system theoretical framework, such as that posed by Romero-Lankao et al., which considers the domains of socio-demographics, economy, technology, environment, and governance in tandem [37]. Even at various scales, it is evident that strategies to dismantle structural inequalities are multisectoral, including the employment, economic empowerment, education, healthcare, housing, criminal justice, and environmental sectors [35].

Primarily building off of Smith et al. and Yearby, our own framework first acknowledges how systems of oppression have influenced policies, practices, and funding. Systems of oppression include ableism, classism, racism, and sexism. Of note, racism was the only system of oppression that was specifically identified in our review of the hurricane-related health vulnerability literature, highlighting significant gaps in scholarship. These systems of oppression perpetuate discriminatory policies, practices, and funding strategies, which in turn impact the social determinants of health, including public health and health care, neighborhood and built environment, education, and economic stability [28]. Subsequently, these social determinants of health create pathways of climate vulnerability at the community and individual levels [27], which ultimately lead to health inequities.

Going beyond existing frameworks, we use the results of our literature review to describe the types of policies, practices, and funding that may be most relevant to climate-related health vulnerability. In addition to racism, the major factors that emerged from the five reviewed studies that included structural determinants of vulnerability were governance, infrastructure, and institutions. Here, we provide further context on how these factors relate to structural determinants and may contribute to health vulnerability from climate-related disasters. While not exhaustive, our conceptual framework provides key examples linking discriminatory policies, practices, and funding to hurricane (and other climate-related event) vulnerability.

#### Policies, Practices, and Funding and Their Links to Climate Vulnerability

##### Governance

The role of governance in hurricane vulnerability is complex due to the interactions between multiple levels of governing bodies and their activities. Organizational and institutional collaborations address disaster governance through norms, laws, regulations, network capabilities, and best practices (e.g., capacity building, early warning systems, planning, insurance arrangements) [38]. The goal of disaster governance is a reduction in disaster-related risks and vulnerabilities, but various contextual factors, such as urbanicity, access to services, political empowerment, disproportionate exposure to hazards, sustainable development, and other sociodemographic trends, can affect the successes and failures of these strategies [38]. Additional challenges for disaster governance involve transparency, debt, siloed efforts, and bureaucracy [39–41].

For example, Hurricane Harvey exposed sources of vulnerability stemming from state governance structures that contributed to higher exposures in the built environment (e.g., weak building code requirements) and increased sensitivity (e.g., self-insured government assets), and coordination with federal actors and policies for disaster assistance complicated the local response [42]. State and national buyout and rebuilding programs for households living in floodplains are also examples of programs that can reinforce pre-existing inequalities depending on how they are designed, as lower-value housing requires sufficient grant funding that is not always available [43, 44].

In addition, democracy and representation are tools that can empower communities and populations before, during, and after a hurricane event. However, severe outcomes (e.g., delays in medical care, lack of emergency and temporary shelter, information gaps between departments and jurisdictions) can occur without the democratic accountability of elected officials and other intersectoral emergency managers such as in the case of levee system failures during Hurricane Katrina where authority of the system was unclear within the governance network [45].

Socially vulnerable populations, including low-income, people of color, and prisoners, often lack political authority, which contributed to perceived powerlessness and lack of citizen accountability, as in the case of Hurricane Katrina [46]. Indicators of less than optimal democracy and representation, including lower voting rates across jurisdictions and levels of government, are also associated with both social vulnerability and a variety of negative health outcomes (e.g., mortality rates, chronic health conditions, hospitalizations, mental health, general well-being) [47]. Access to social capital and life-saving services such as health insurance may mediate the relationship between voting and health [47, 48] and may also explain why some populations are more resilient in the face of climate-related disasters [49, 50]. These social determinants frequently lead to more severe post-hurricane health outcomes. Immigrant status is another factor that can limit representation and lead to increased vulnerability and more severe hurricane-related outcomes [51].

##### Infrastructure

In the case of infrastructure, residential segregation has marginalized low-income and communities of color into neighborhoods that are at greater risk of experiencing climate and other environmental hazards through zoning and housing policies, contributing to health disparities [52–55]. For example, historical redlining has shaped the urban landscape in communities across the country. Areas that were previously redlined (e.g., with higher-risk Home Owners’ Loan Corporation grade scores) have been associated with greater present-day flood risk and heat exposure, and these communities also have limited financial and environmental capital to mitigate these climate risks [53, 56, 57]. Similar codified laws such as discriminatory Jim Crow laws, along with non-codified predatory home lending practices, are other examples of policies that gave rise to unequal neighborhood and built environment conditions for racially and ethnically minoritized communities [58]. These communities, therefore, have stark inequalities in disaster preparedness and recovery due to disinvestment in their neighborhoods and investment in white and wealthy neighborhoods (e.g., more flood-protection funds for areas with higher property values) [56]. Additionally, these laws and redlining practices created disparities in access to green space, which is an important permeable surface and buffer for flooding and the urban heat island effect [58–60]. This association between redlining and lower access to greenspace was examined in a spatial analysis of Indianapolis, along with higher incidence of toxic waste sites [61]. The health impacts from hurricanes are exacerbated when flooding occurs in environmental justice communities, which are also home to large numbers of polluting sources, as floodwaters can carry contaminants into proximity of residential areas [11, 62].

Infrastructure not only impacts the magnitude of exposure or impact of an extreme weather-related event but can also slow down response and recovery. For example, low-income communities have a slower recovery, often due to delays in insurance payouts, loss of family income, prolonged loss of infrastructure services, and housing and rental recoveries [63–65]. Additionally, reliability of the power grid varies across the United States, and failures often disproportionately affect marginalized and underserved neighborhoods due to historic disinvestment and current management practices, increasing social vulnerability of the population residing there. For example, during the Texas winter storm of 2021, data indicate that the extent and duration of managed power outages was greater in areas with low-income and racial/ethnic minoritized populations [66]. Similar patterns have been seen with hurricanes, such as during Hurricane Irma, when the spatial distribution of electric power outages was concentrated in areas with greater minoritized, disabled, and economically vulnerable populations [63]. The interdependencies of infrastructure systems make hurricane-related damage to their services especially precarious. For example, electric power outages and associated distribution system failures, although have a low-probability of occurrence, result in high impact damage, and can disrupt other essential services and resources in places such as hospitals [64, 67]. Other examples of interdependent, infrastructure-related policies, practices, and funding that impact hurricane vulnerability include transportation, utilities, healthcare, and communications [68–71].

##### **Institutions** 

Institutions such as educational and prison systems can create barriers depending on neighborhood and community socioeconomic characteristics. Education is an upstream driver of health, and the quality of education can influence its role in disaster risk reduction and providing a normal environment for a child during and after a hurricane event [72, 73]. However, weak infrastructure and other systemic factors may contribute to delayed school reopenings such as the case with Hurricane Katrina [72]. Education also impacts employment (which has implications for health insurance, financial stability, and the ability to evacuate) and where you can afford to live (which influences exposures to environmental hazards) in a generationally repetitive cycle [74].

Additionally, the incarcerated population is often overlooked, particularly in policies related to disaster preparedness and evacuation plans. For example, there was no evacuation plan for the Orleans Parish Prison during Hurricane Katrina, causing a lockdown of thousands of inmates, which led to severe hunger, disease, violence, exposure to contaminated floodwaters, and mortality [75]. Prison systems also tend to have aging infrastructure and poor physical conditions (e.g., overcrowding) that are worsened during climate events, extended to non-hurricane events as well. An analysis of U.S. state and private prisons revealed an association between warm temperatures and mortality, particularly for heart-disease related mortality and suicide [76]. In Texas, it was found that heat-related deaths had a greater association with prisons without air conditioning compared to those with air conditioning [76]. As the incarcerated population continues to grow, along with the unequitable distribution of the racially minoritized and low-income that constitutes this population, public health interventions like widespread air conditioning policies require an institutional-level approach to ameliorate such harsh conditions [77].

Religious institutions can also impact health outcomes during and immediately following hurricanes. Religious coping mechanisms, such as spirituality and religious participation, have been associated with population mental health outcomes and overall resilience throughout the disaster recovery process during and post-Hurricane Katrina [78, 79]. These mechanisms can augment social support, cognitive processing of a traumatic event, and personal empowerment and attachment to the hurricane event [80]. Additionally, faith-based organizations can be a vital resource for community members that increase social cohesion and offer social support and direct services (e.g., provision of food, shelter, childcare, and other resources during and after evacuation periods), both of which can be mutually reinforcing [78, 80]. Despite the critical role that religious institutions play in hurricane relief, their expertise and network are often not leveraged for disaster policies [78]. There are opportunities, therefore, to remove siloes between government, nonprofit, and other organizations and formulate more effective hurricane preparedness plans.

#### Cascading Events and Repeated Events

Finally, our conceptual framework accounts for cascading and repeated events. Cascading events can also pose additional hazards in the pathways of hurricane-related vulnerability. These cascading hazards occur as a direct or indirect result of the initial hurricane event within a proximal spatiotemporal period [81]. Such hazards are closely tied to multiple infrastructure system disruptions, delayed restoration efforts, and ecosystem disturbances [82, 83]. For example, several restoration interdependencies were identified post-Hurricane Sandy, where a restoration task in one infrastructure system hindered the initiation, effectiveness, time-sensitivity, or the availability of options or resources for another task in a different infrastructure system (e.g., residential, power, telecommunications, emergency services, hospital, subway sectors) [84].

Repeated hurricane and extreme precipitation events also occur within a short period of time, such as with Hurricanes Maria and Irma in Puerto Rico and two one-in-1,000 year flooding events in Ellicott City, Maryland. These repeated events can stretch the limits of even well-resourced human and resource capital. For example, when Harris County, Texas experienced repeated flooding before Hurricane Harvey, the hospitals had challenges with staff burnout and transportation despite having advanced emergency preparedness due to this prior experience [85]. Thus, it is critical to account for secondary events (both cascading and repeated) when considering hurricane-related health vulnerability.

### Recommendations for Future Research

We reviewed the literature on vulnerability to hurricane-related health impacts over the last two decades. Traditional methods characterizing socially vulnerable populations have been useful to determine how hurricanes and other climate disasters impact certain communities differently. However, as shown in our results, there is now a preponderance of literature establishing these characteristics and which populations are affected most.

Prior reviews of hurricane vulnerability focus on spatial impact modeling of physical [86] or current measures of social vulnerability (e.g., CDC Social Vulnerability Index) [87]. Few studies assess upstream, structural determinants of vulnerability, although doing so could provide important information on causes of causes and potential levers for policy change. Although structural determinants are not commonly accounted for in studies of hurricane health vulnerability, our broader review of the literature indicates that they may play a key role in vulnerability to the health impacts of hurricanes and other climate-related disasters.

Going forward, we recommend that hurricane and climate-related vulnerability assessments capture structural determinants of health to better characterize the upstream drivers of health outcomes and inform opportunities for public health interventions. Although future research will be necessary to more appropriately elucidate structural determinants within the climate hazard context, we provide examples of several indicators (Table 1) that can be used to capture the domains described in our conceptual framework. While these factors emerged from our review of literature related to hurricane vulnerability, these measurements may also be useful for other climate stressors, such as heatwaves and wildfires.

**Table 1:** Example indicators for measuring structural determinants of hurricane vulnerability

Structural Determinant	Upstream Driver	Data Source	Data Source Description	Link/ Source/ Software	Granularity	Tie to Climate/ Hurricanes
Governance	Voting/ Representation	Cost of Voting Index [88]	State-level measure of difficulty in voting during an election, using data from 1996 to 2016	https://costofvotingindex.com/data	State	Hurricanes can displace voters and exacerbate existing structural barriers such as transportation to voting locations and restrictions on absentee ballots [89].
		National Conference of State Legislatures [90]	Legislature % Black^b^: percent of legislators who identify African American/ Black as their race/ ethnicity	https://app.powerbi.com/view?r=eyJrIjoiOWM4YTI4YjQtZDM2Yy00YWU5LTkyNGYtODYxMmUxZTExNDc3IiwidCI6IjM4MmZiOGIwLTRkYzMtNDEwNy04MGJkLTM1OTViMjQzMmZhZSIsImMiOjZ9	State	
	Immigration	Internal Displacement Monitoring Centre (IDMC), United States Profile [91]	Internal Displacements: preliminary estimates of internal displacement events reported in the last 180 days	https://www.internal-displacement.org/countries/united-states	National	Immigrant status is associated with increased hurricane vulnerability through lower levels of self-protection and hazard knowledge, and higher perceptions of risk [92]. Reconstruction efforts are often seen as job opportunities for disaster migrants, with a large proportion of this population being undocumented Latinx immigrants [93].
		U.S. Census Bureau, Foreign Born Data [94]	Foreign Born: socioeconomic and demographic characteristics of the foreign born population by census tract	https://www.census.gov/topics/population/foreign-born/data.html	Census tract	Data on foreign born populations have been examined for demographic and socioeconomic characteristics in states affected by hurricane disasters [95].
	Civics	U.S. Census Bureau, Decennial Census of Population and Housing, Operational Quality Metrics [96]	Census Return Rate^b^: measure of self-response among all addresses in the Census	https://www.census.gov/programs-surveys/decennial-census/decade/2020/planning-management/count/response-rates.html	County	Hurricanes and disaster planning and recovery often spur civic engagement, especially to represent vulnerable communities that may experience delays and disparities in response efforts [97].
Infrastructure	Residential segregation	The Eviction Lab, National Zoning and Land Use Database (NZLUD) [98]	Exclusionary zoning: condenses land use policies into a single measure of exclusionary zoning (Zoning Restrictiveness Index (ZRI))	https://github.com/mtmleczko/nzlud	Metropolitan area	Exclusionary zoning is a form of residential segregation that marginalizes racially minoritized communities by allowing for a disproportionate exposure to toxic chemical sites, especially during hurricane scenarios [99].
		Boston College Department of Economics [100]	Diversity Index (Housing), aspatial^a^: measures the extent to which several groups (i.e., racially/ ethnically minoritized groups) are present in a metropolitan area, regardless of their distribution across census tracts	https://econpapers.repec.org/software/bocbocode/s375001.htm	Metropolitan area	Post-hurricane housing recovery is largely influenced by the type of housing and other neighborhood socioeconomic characteristics [101, 102].
		U.S. Census Bureau, American Community Survey [103]	Dissimilarity Index (Housing)^a^: measure of the evenness of how Black and white residents are distributed across census tracts that make up a larger geographic area	https://www.census.gov/topics/housing/housing-patterns/guidance/appendix-b.html	Census tract	Regions with a high proportion of low-income communities and communities of color experience more environmental harms and toxic releases, which is exacerbated for communities with pre-existing disaster risk [104].
		U.S. Census Bureau, American Community Survey [105]	H (entropy) Index (Housing)^a^: measure of “evenness” of the extent to which groups (i.e., racially/ ethnically minoritized groups) are evenly distributed among organizational units	https://www.census.gov/topics/housing/housing-patterns/about/multi-group-entropy-index.html	Metropolitan area	
		U.S. Census Bureau [106]	Index of Spatial Proximity^a^: mean intragroup proximity for majority and minority groups, weighted by each group’s percentage of the total population	https://www.census.gov/data/tables/time-series/demo/housing-patterns/housing-patterns-tables.html	Metropolitan area	
		University of Richmond Mapping Inequality [107]	Historical Redlining: scores from the Home Owners’ Loan Corporation (HOLC) maps, graded into four categories (i.e., from A, “best” to D, “hazardous”	https://dsl.richmond.edu/panorama/redlining/#loc=5/39.1/−94.58	Census block group	Historically redlined neighborhoods face a greater risk of flooding compared to their counterparts [56, 108].
	Healthcare	Centers for Disease Control and Prevention, Compressed Mortality File [109]	Mortality ratio^a^: Age-adjusted non-Hispanic black to non-Hispanic white mortality rate	https://www.cdc.gov/nchs/data_access/cmf.htm	State and county	Tropical cyclones are associated with higher county-level mortality rates, and a greater proportion of low-income communities and communities of color live in tropical cyclone-affected areas [110].
		U.S. Census Bureau [111]	Insurance ratio^a^: Ratio of proportion of white non-Hispanic to black county residents with health insurance	https://www.census.gov/data/tables/2022/demo/health-insurance/p60-278.html	National	Unemployment following a hurricane can constrain access to health care [112].
		Dartmouth Atlas of Healthcare [113]	Primary care ratio^a^: Ratio of average annual proportion of white non-Hispanic to black non-Hispanic Medicare enrollees having at least one ambulatory visit to a primary care clinician	https://www.dartmouthatlas.org/	County	Hurricane-related flooding may be associated with declines in health care utilization, with disparities by flood status, race, and income [112, 114].
		Centers for Disease Control and Prevention [115]	Flu vaccination by race^b^: Percentage vaccinated by month	https://www.cdc.gov/flu/fluvaxview/dashboard/jurisdiction-IIS-coverage.htm	State	
		County Health Rankings & Roadmaps [116]	Preventable hospitalization by race^b^: Rate of hospital stays for ambulatory-care sensitive conditions per 100,000 Medicare enrollees	https://www.countyhealthrankings.org/explore-health-rankings/county-health-rankings-model/health-factors/clinical-care/quality-of-care/preventable-hospital-stays? keywords=21205&f%5B0%5D=type%3Astates&f%5B1%5D=type%3Acounties&year=2023	County, State, or Zip Code	
		Health Resources and Services Administration, Bureau of Health Workforce [117]	Health Professional Shortage Area (HPSA) Score^b^: score designated by the National Health Service Corps (NHSC) for determining priorities for assignment of clinicians, ranging from 0 to 26	https://data.hrsa.gov/tools/shortage-area/hpsa-find	County	
	Utility shut-offs and power outages	U.S. Energy Information Administration [118]	System Average Interruption Duration/ Frequency Index, Customer Average Interruption Duration Index	https://poweroutage.us/	County	Socially vulnerable communities have been found to experience a greater burden of power outages associated with climate events [119]. This is especially concerning for medically fragile patients using electricity-dependent durable medical equipment.
	Flood insurance and hazard mitigation	Federal Emergency Management Agency (FEMA) [120]	OpenFEMA Datasets: publicly available data on FEMA’s disaster information, alerts, individual assistance, public assistance, hazard mitigation, and National Flood Insurance Program (NFIP)	https://www.fema.gov/about/openfema/data-sets	Household	Flood insurance and hazard mitigation assistance has largely favored white and affluent households due to requirements for this grant money [121].
Institutions	Education system	U.S. Census Bureau, American Community Survey [122]	College graduation ratio^a^: Ratio of non-Hispanic white to black college degree prevalence	https://www.census.gov/topics/education/educational-attainment.html	Census tract	Hurricane Katrina led to the diaspora of African American college students and delayed recovery of Historically Black Colleges and Universities (HBCUs), creating long-term impacts on higher education for these students [123]. Similar impacts can occur in other hurricane contexts.
		National Student Clearinghouse Research Center [124]	Historical college completion by race^b^: completion rates in American higher education	https://nscresearchcenter.org/signaturereport12-supplement-2/	Educational institution	
	Education system (school segregation)	U.S. Department of Education [125]	School dissimilarity index^a^: contribution of individual schools to the racial and ethnic segregation of US school districts	https://datacatalog.urban.org/dataset/index-school-contribution-racial-segregation-us-school-districts	Census tract	Post-hurricane school reforms may be associated with school segregation by race/ ethnicity and other socioeconomic factors [126].
		U.S. Department of Education [127]	Thiel’s H index^a^: measure the degree to which racial and ethnic groups within a congressional district are evenly distributed across its census tracts	https://www2.census.gov/programs-surveys/demo/about/housing-patterns/multigroup_entropy.pdf		
		American Communities Project, Brown University [128]	School segregation lawsuits^b^: segregation indices for cases in a state	https://s4.ad.brown.edu/Projects/USSchools/SchoolDLoad.htm	School district	
	Prison systems/ criminal justice	U.S. Department of Justice, Census of Jail Inmates, 2005 [129]	Jail incarceration ratio^a^: ratio of non-Hispanic black to non-Hispanic white county jail incarceration	https://bjs.ojp.gov/library/publications/jail-inmates-2021-statistical-tables	National and state	People of color, less well-educated, lower-income, and those with chronic and communicable disease represent a disproportionate amount of the American incarcerated population [77]. As mass incarceration continues to grow, prisoners are faced with poor living conditions from overcrowding and aging infrastructure, which are exacerbated under extreme climate scenarios.
		U.S. Census Bureau, Decennial Census 2000 [130]	Jail incarceration ratio^a^: ratio of black to white county jail incarceration	https://www.census.gov/econ/overview/go3000.html		
Racism/ Classism	Income inequality	Opportunity Insights, Social Capital Atlas, Harvard University [131]	Generational income mobility^b^: measure of wage persistence across generations	https://socialcapital.org/	County, Zip code	Hurricanes create large income shocks to households, with greater impacts for lower income households who are more vulnerable due to housing, insurance, and employment disparities [132].
		U.S. Census Bureau, Race and Economic Opportunity Data Table [133]	Racial income inequality^b^: de-identified data on racial gaps across generations	https://www.census.gov/programs-surveys/ces/data/public-use-data/race-and-economic-opportunity-data-tables.html	Census tract	

^a^ Data sources have been previously studied in Dougherty et al. as potential components of an aggregated structural racism score [134]^b^ Data sources have been previously studied in LaFave et al. as measures of structural racial discrimination [135]

It should be noted that relevant non-health impacts (e.g., evacuations) were not captured in this review of literature due to the exclusion of articles without health outcome assessments. However, future studies examining structural determinants of hurricane vulnerability should also consider non-health impacts. Additionally, not all of the indicators listed in Table 1 will be available at the appropriate level of granularity to conduct vulnerability assessments of the health impacts of climate-related stressors (e.g., state-level Cost of Voting Index). Thus, future research is needed to refine and expand upon this list of indicators.

## Conclusions

Upon reviewing prior literature on vulnerability to hurricane-related health outcomes, the vast majority have focused on demographic and socioeconomic factors as the major drivers of associated health disparities. Upstream structural determinants, such as governance, infrastructure, institutions, and systemic racism can increase climate vulnerability but have been understudied. We provide a conceptual framework to explore examples of the links between systems of oppression, structural determinants of health, pathways of vulnerability, and health disparities. We also recommend incorporating data sources related to these structural determinants into climate and health vulnerability studies to inform public health interventions that improve health equity, climate resilience, and adaptive capacity.

### Key References

- Rodriguez-Díaz CE, Lewellen-Williams C. Race and Racism as Structural Determinants for Emergency and Recovery Response in the Aftermath of Hurricanes Irma and Maria in Puerto Rico. Health Equity [Internet]. 2020 May 22;4(1):232–8. Available from: https://www.ncbi.nlm.nih.gov/pmc/articles/PMC7247035/**This paper identifies structural racism as a structural determinant of hurricane vulnerability.**
- Tennant E, Gilmore EA. Government effectiveness and institutions as determinants of tropical cyclone mortality. Proc Natl Acad Sci U S A. 2020 Nov 17;117(46):28,692–9. **This study identifies governance as a structural determinant that influences tropical cyclone mortality.**
- Dement C, McAleavy T. Vulnerable populations: A cross-case synthesis of correctional facility disaster response during Hurricanes Katrina and Maria. J Emerg Manag. 2022 Special Issue on Puerto Rico;19(8):97–108. **This cross-case synthesis study highlights the prison system as an institution**,** which is a structural determinant of hurricane vulnerability.**
- Guerra Velázquez GR. Hurricane María and Public Health in Puerto Rico: Lessons Learned to Increase Resiliency and Prepare for Future Disasters. Ann Glob Health. 2022;88(1):82. **This paper explored infrastructure deficiencies as a structural determinant of hurricane vulnerability.**
- Ruiz-Aviles VD, Pijawka D, Manuel-Navarrete D, White D, Ortiz-Garcia C. Restoration versus transformative adaptation of community drinking water systems after Hurricanes Irma and Maria in Puerto Rico. J Emerg Manag. 2022 Special Issue on Puerto Rico;19(8):25–40. **This study investigated community drinking water systems**,** which is an infrastructure deficiency that can be identified as a structural determinant of hurricane vulnerability.**
- Smith GS, Anjum E, Francis C, Deanes L, Acey C. Climate Change, Environmental Disasters, and Health Inequities: The Underlying Role of Structural Inequalities. Curr Envir Health Rpt [Internet]. 2022 Mar 1;9(1):80–9. Available from: 10.1007/s40572-022-00336-w/. **This review provides a conceptual framework for environmental disaster health inequities.**
- Yearby R. Structural Racism and Health Disparities: Reconfiguring the Social Determinants of Health Framework to Include the Root Cause. J Law Med Ethics. 2020 Sep;48(3):518–26. **This article presents a framework describing how structural racism contributes to social determinants of health**,** which addresses the underlying causes of racial health disparities.**

## Supplementary Information

Below is the link to the electronic supplementary material.

ESM1 (XLSX 103 KB)

## Data Availability

No datasets were generated or analysed during the current study.
